# Wettability Study of Soldered Joints in SiC Ceramics and Combined Ni-SiC Using SnSbTi-Based Solder and Electron Beam Heating

**DOI:** 10.3390/ma18122814

**Published:** 2025-06-16

**Authors:** Tomas Melus, Roman Kolenak, Jaromir Drapala, Peter Gogola, Matej Pasak, Daniel Drimal, Mikulas Sloboda

**Affiliations:** 1 Faculty of Materials Science and Technology in Trnava, Slovak University of Technology in Bratislava, Jána Bottu n. 2781/25, 917 24 Trnava, Slovakia; roman.kolenak@stuba.sk (R.K.); peter.gogola@stuba.sk (P.G.); matej.pasak@stuba.sk (M.P.); mikulas.sloboda@stuba.sk (M.S.); 2 FMT—Faculty of Materials Science and Technology, Technical University of Ostrava, 17. Listopadu 15, 708 33 Ostrava, Czech Republic; jar.drapala@seznam.cz; 3 The First Welding Company Inc., Kopčianska 14, 851 01 Bratislava, Slovakia; drimal.daniel@pzvar.sk

**Keywords:** wettability, soldered joints, SiC ceramics, electron beam heating, Sn-Sb-Ti solder, Ni-SiC composite, reaction layer, shear strength, diffusion

## Abstract

The reactive soldering of silicon-carbide (SiC) ceramics to a Ni-SiC composite was investigated using an Sn-5Sb-3Ti active solder and electron-beam heating at 750 °C, 850 °C and 950 °C. Wettability: The average contact angle decreased from 94 ± 4° (750 °C) to 60 ± 3° (850 °C) and further to 24 ± 2° (950 °C), demonstrating progressively improved spreading of the filler with increasing temperature. Interfacial reactions: Continuous layers of Ni_3_(Sn,Sb)_4_ and Ti_6_(Sn,Sb)_5_ formed along the Ni-SiC/filler interface, the latter confirming Ti diffusion that activates the wetting of the composite surface. Mechanical performance: Shear-lap tests on three joints per condition yielded 39 ± 6 MPa (750 °C), 27 ± 2 MPa (850 °C) and 36 ± 15 MPa (950 °C). The highest and lowest individual values at 950 °C were 51 MPa and 21 MPa, respectively. These results show that a higher soldering temperature lowers the contact angle and promotes interfacial reaction, but only a moderate improvement in average joint strength is obtained. These findings demonstrate a flux-free route to bond SiC ceramics with Ni-SiC composites, which is highly relevant for next-generation power-electronics modules and other high-temperature applications.

## 1. Introduction

SiC ceramics belong to a group of materials which are characterised by their excellent chemical, physical, and mechanical properties, making them suitable for high-temperature applications. SiC ceramics exhibit good strength at elevated temperatures and excellent corrosion resistance. Therefore, SiC finds broad applications, mainly for parts in power semiconductors, as its properties are suitable for this purpose as well as being cheap and easily accessible [1,2,3,4,5,6].

For these applications, SiC ceramics often have to be bonded to various metals to create composite structures; however, mismatched thermal expansion coefficients can complicate the joining process and frequently compromise the resulting joints.

For many high-temperature or electronic applications, SiC ceramics have to be joined to metals. However, the large mismatch in thermal expansion coefficients and the tendency to form brittle reaction products such as silicides or carbides make reliable metal–SiC bonds difficult to achieve [7].

The nickel-based composites exhibit excellent properties, such as high resistance against corrosion or wear. Therefore, they find applications in a broad range of fields, including the electrotechnical, automotive, and aviation industries. Nanoparticles are frequently used as a reinforcing phase in nickel-based composites, to increase their microhardness or wear and pyro-oxidation resistance. Ni-based composites are generally prepared via the processes of powder metallurgy [8,9,10,11].

However, commercial soldering alloys are not suitable for joining such materials, since they do not react efficiently with the ceramic or composite substrates. This issue can be solved by soldering using alloys containing active elements, such as Ti, Zr or, V, which allows for excellent chemical reactions with those substrates. The active soldering alloys are able to bond efficiently with the ceramic surface, owing to reaction products formed between the active element and ceramic or composite substrates [12,13,14,15,16,17,18,19].

Active-element additions typically range from 2 to 4 wt %, a level that can considerably raise the solder alloy’s melting temperature. To join ZrO_2_ ceramics with 304 stainless steel [12], we employed a micro-alloyed active solder (Sn-3Ag-0.5Cu-0.2Ti). Ultrasonic soldering promoted an intense interaction between the filler and the base materials, generating Zr_0.5_Ti_0.35_Sn_0.15_O_2_ and Ti_11.3_Sn_3_O_10_ phases along the solder/ZrO_2_ interface. The resulting joints reached a peak shear strength of roughly 31 MPa.

In [14], the bonds between the active soldering alloy type SnAg_3.5_Ti_4_ and SiO_2_ were studied. The experimental results suggest that the apparent segregation of Ti and the formation of the TiSi and TiO_2_ phases occurs on the solder/substrate boundary. Ti found on the boundary with the SiO_2_ substrate plays a significant role, primarily in the initial phase of bond formation. The shear strength of SiO_2_/SiO_2_ joints attained an average value of 15 MPa.

In our previous study, we used SnSb5Ti3 filler to investigate the wetting and soldering of SiC ceramics during electron beam heating in a vacuum. The alloy operates in a narrow melting range and reaches a liquidus temperature of approximately 243 °C [20,21,22,23,24].

It was observed that the wetting angle on the SiC ceramic substrate decreased with increasing temperature. The best results for wettability were achieved at a temperature of 950 °C, where the wetting angle was 33°. The formation of the TiSi_2_ and Ti_3_Ni_5_Si_6_ (and Ni_3_Sn_2_ and Ni_3_Sn) phases were observed on the boundary with SiC ceramics. The highest shear strength, around 40 MPa, was achieved at the soldering temperature of 850 °C.

Building on our earlier investigations of Sn-Sb-Ti fillers for ultrasonic soldering [24] and electron-beam soldering [25], this study focuses on the wettability and shear strength of combined SiC-ceramic/Ni-SiC joints produced with Sn-5Sb-3Ti active solder under electron-beam heating in vacuum. The electron beam delivers the required thermal energy within seconds, raising the joint temperature above ~700 °C—the threshold for activating titanium in the filler—while minimising overall soldering time. The coefficient of thermal expansion (CTE) of hexagonal SiC is 4.2 × 10^−6^ K^−1^; for our Ni–SiC composite (40 vol % SiC in Ni) it is 8.5 × 10^−6^ K^−1^; and for the Sn–5 wt % Sb–3 wt % Ti solder it is 22.5 × 10^−6^ K^−1^. These mismatches in CTE during heating to 950 °C can induce stresses at the solder/substrate interface, underscoring the importance of forming a graded intermetallic layer to relieve thermal-expansion-mismatch stresses.

## 2. Materials and Methods

After establishing the target mass ratios, each alloy constituent was weighed individually. Feedstock of 4 N purity served as the starting material for the solder. The charge was then melted in an induction vacuum furnace, with the pre-weighed components placed inside an Al_2_O_3_ ceramic crucible. The experiment was carried out under flowing argon (grade 4.6, 99.996% Ar; ISO 14175 [26]), at a pressure of 200 Mbar. Solder synthesis was performed at roughly 1100 °C, a temperature that allowed titanium to dissolve gradually into the molten alloy. The elemental composition of the resulting solder was established experimentally and is reported in Table 1. The chemical composition of the soldering alloy was analysed using the method of atomic electron spectrometry with induction coupled plasma (ICP-AES). The analysis was performed using the SPECTRO VISION EOP instrument (SPECTRO Analytical Instruments, Kleve, North Rhine-Westphalia, Germany), where samples of the alloys were dissolved in suitable chemical solutions of acids and bases. Elemental analysis was carried out on an emission atomic spectrometer fitted with a pneumatic nebuliser and a Scott-type spray chamber.

**Figure 1:**
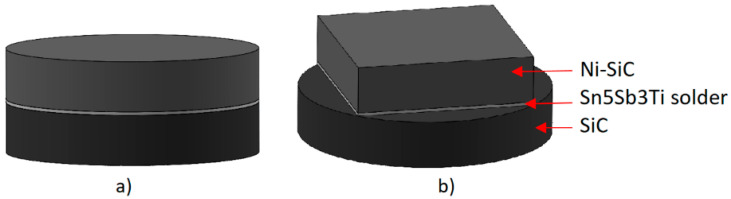
Preparation of soldered joints: (**a**) specimen designated for characterising the solder–substrate interface; (**b**) specimen prepared for shear-strength testing.

The experimental programme employed the substrates and filler metals depicted in Figure 1. Sn5Sb3Ti solder was machined into 4 × 4 × 4 mm cubes for both wettability testing and joint fabrication. The SiC ceramic substrate was prepared as circular specimens 15 mm in diameter and 3 mm thick, whereas the Ni–SiC composite substrate was produced either as discs of identical size or as square plates measuring 10 × 10 × 3 mm. Prior to this, all substrates were ultrasonically cleaned in acetone for 10 min and then in ethanol for 10 min, and finally dried at 60 °C for 1 h. Before electron-beam soldering, the filler alloy was ultrasonically pre-deposited onto the substrate surface. This procedure is discussed in [20].

**Figure 2:**
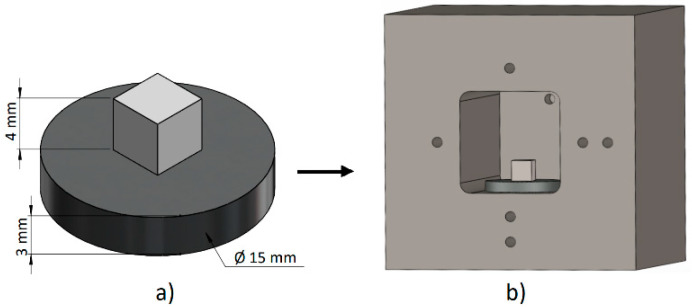
Schematic representation of (**a**) Ni-SiC + SnSb5Ti3; (**b**) layout of substrate/solder system inside the jig.

The joints were produced in a vacuum electron-beam soldering system. The soldered specimens were placed in a special graphite jig, as shown in Figure 2b, which was located in a vacuum chamber. The defocused electron beam hit the graphite jig, which resulted in its heating. Through the radiant heat from the jig, secondary heating of the specimens took place. The temperature during the entire soldering process was monitored via thermocouples located in the jig openings. The same heating method was applied for the soldered joints and also for the wettability specimens.

**Figure 3:**
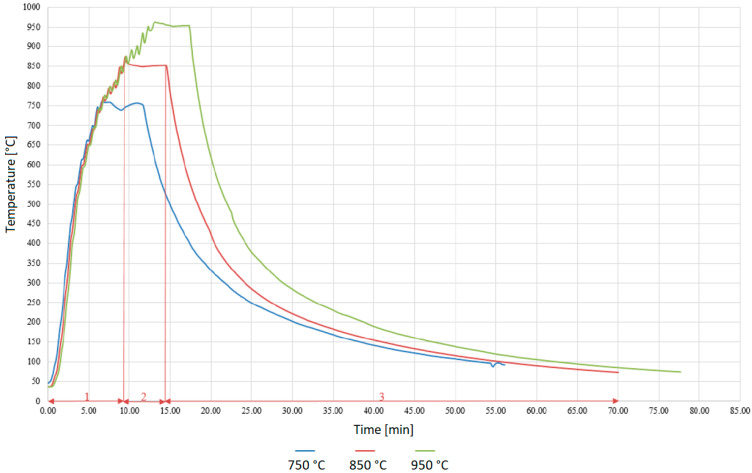
The soldering thermal profile comprised three stages: 1—heating phase, rate 90 °C/min; 2—holding phase during 5 min; 3—cooling phase, rate 15.6 °C/min.

Figure 3 shows the working cycles for the temperatures employed in the experiment. This consisted of a rapid heating up to a desired soldering temperature, in our case 750, 850 and 950 °C, with an approximate heating rate of 90 °C/min. This was followed by a dwell time of 5 min at the soldering temperature and subsequent slow cooling at an approximate rate of 15.6 °C/min. The temperature set-points were selected with reference to earlier work [21], which evaluated the wettability of an Al_2_O_3_ ceramic substrate using a Sn-based solder containing 2 wt % Ti. That investigation showed that the effective activation of titanium requires temperatures of at least 750 °C.

The complete set of electron-beam soldering parameters is listed in Table 2.

The metallographic preparation of specimens from soldered joints was performed using standard metallographic preparation procedures. Grinding was performed using SiC emery papers with 240, 320, and 1200 grains/cm^2^ granularity. Polishing was performed with diamond suspensions with grain sizes of 9 μm, 6 μm, and 3 μm. Final polishing was performed with a colloidal-silica suspension OP-S (Struers ApS, Ballerup, Denmark) having a grain size of 0.2 µm. The solder microstructure was studied through the use of scanning electron microscopy (SEM) on the TESCAN VEGA 3 microscope. To perform qualitative and semi-quantitative chemical analysis, the JEOL 7600 F with an X-ray micro-analyser type Microspec WDX-3PC (SEM/EDX, JEOL Ltd., Tokyo, Japan) was used. X-ray diffraction analysis was used to identify the phase composition of the solder. It was carried out on a 10 × 10 mm solder sample on a PANalytical X’Pert PRO XRD diffractometer (Malvern Panalytical Ltd., Malvern, UK). X-ray diffraction (XRD) measurements were performed using a PANalytical Empyrean diffractometer (Malvern Panalytical Ltd., Malvern, UK) equipped with a Cu X-ray tube (λ_Kα1_ = 1.5406 Å) set at 40 kV/40 mA. Diffraction data were collected using a PIXcel3D area detector (Malvern Panalytical Ltd., Malvern, UK) operated in scanning mode. The measurement covered a 2θ range from 20.0° to 130.0°, with a continuous scan and a counting time of ~38 s per step. The patterns were evaluated using the PANalytical Xpert High Score program (HighScore Plus 3.0.5 version) with the ICSD FIZ Karlsruhe database.

**Figure 4:**
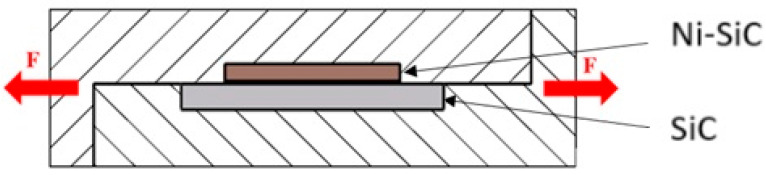
Scheme of shear strength measurement [20].

The mechanical properties of soldered joints were determined by the shear strength test. The schematic representation of this test is documented in Figure 4. The shear strength was measured using the LabTest 5.250SP1-VM (LABORTECH s.r.o., Opava, Czech Republic) located at the Faculty of Materials Science and Technology, Slovak University of Technology (MTF STU), Trnava, Slovakia. A jig with the defined shape of the test specimen was used for the change in orientation of the loading force. The purpose of this shearing jig was to guarantee consistent loading of the specimen, achieving shear within the plane of the solder/substrate boundary.

## 3. Results

### 3.1. Wettability and Interaction of Sn5Sb3Ti Solder on the Surface of Composite Material Type Ni-SiC

**Figure 5:**
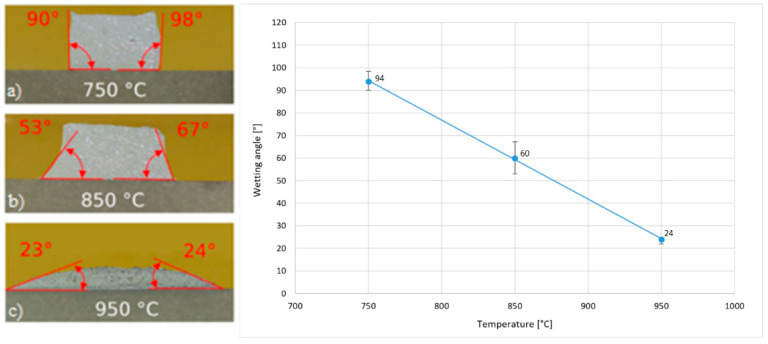
Wettability experiment of Sn5Sb3Ti solder on a composite Ni-SiC substrate at different temperatures: (**a**) 750 °C; (**b**) 850 °C; (**c**) 950 °C.

The sessile-drop wettability test was first conducted at 750 °C. At this temperature, the Sn5Sb3Ti solder remained almost spherical and the contact angle was 94 ± 4°, i.e., non-wetting. Raising the temperature to 850 °C activated titanium in the filler and lowered the angle to 60 ± 3°. A further increase to 950 °C produced pronounced wetting, with the angle falling to 24 ± 2°. This monotonic decrease in contact angle with soldering temperature is summarised in Figure 5: Figure 5a–c shows the droplet images at 750, 850 and 950 °C, while Figure 5c plots the contact angle as a function of temperature. Note that at 750 °C, with the 30 s dwell used, the Sn5Sb3Ti block did not fully melt and remained essentially solid; hence, the reported contact angle of 94 ± 4° reflects a non-equilibrium, qualitative state rather than an equilibrium wetting angle.

**Figure 6:**
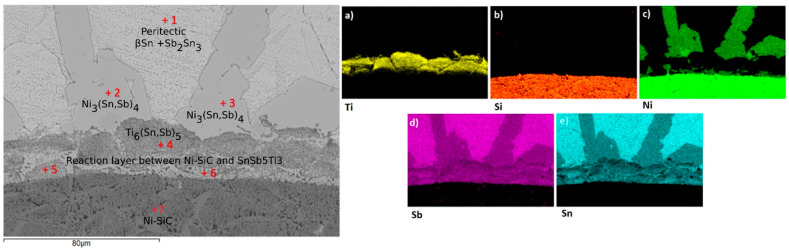
Back-scattered SEM image of the Sn-5Sb-3Ti/Ni-SiC joint produced at 950 °C, overlaid with EDS analysis points 1–7. Elemental maps show the distribution of (**a**) Ti, (**b**) Si, (**c**) Ni, (**d**) Sb and (**e**) Sn. Compositions for points 1–7 are listed in Table 3.

Figure 6 combines a back-scattered SEM image of the Sn-5Sb-3Ti/Ni-SiC interface produced at 950 °C with elemental maps for Ti, Si, Ni, Sb and Sn (Figure 6a–e) and the positions of EDS spots 1–7. The continuous reaction layer at the solder/substrate boundary is strongly enriched in Ti (Figure 6a), showing that the active titanium in the filler is chiefly responsible for wetting the Ni-SiC composite. Local Ni enrichment visible in Figure 6c) indicates a partial diffusion of Ni from the substrate into the layer. Quantitative point EDS data for spots 1–7 are summarised in Table 3. Spectrum 1 corresponds to the peritectic mixture β-Sn + Sb_2_Sn_3_; spectra 2 and 3 originate from Ni_3_(Sn,Sb)_4_ in the solder matrix. Spectrum 4 identifies Ti_6_(Sn, Sb)_5_ inside the reaction layer, whereas spectra 5 and 6 represent heterogeneous boundary zones that contain ≈3 wt % Ti together with Sn, Sb and traces of Ni—a composition that promotes wetting. Spectrum 7 confirms the composition of the bulk Ni-SiC substrate. The microstructure of the Sn5Sb3Ti solder prior to the soldering process, including SEM imaging and phase identification, has been detailed in our previous study [25], and is therefore not repeated in this work.

**Figure 7:**
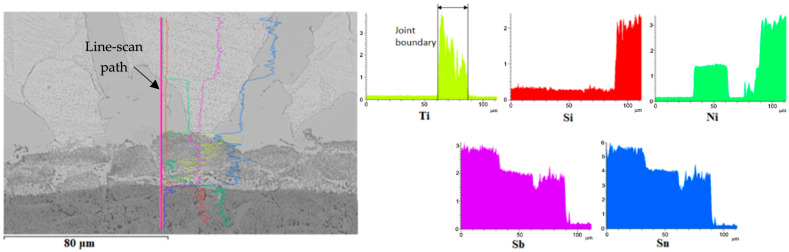
The line EDX analysis of wettability specimen on the Sn5Sb3Ti/Ni-SiC boundary with the given concentration of elements.

Finally, line analysis was performed on the wettability specimen. This is shown in Figure 7 and it represents the precise concentrations of elements in the individual sections marked with the colour lines. This analysis proves that the active Ti element is precipitated on the boundary of the composite Ni-SiC substrate.

The final results suggest that the mechanism of bond formation takes place during the process of electron beam soldering in a vacuum, whereby the active Ti element is distributed to the boundary of the composite Ni-SiC substrate by a diffusion mechanism. Due to this effect, a reaction layer is formed which ensures the wettability of the Ni-SiC substrate. The approximate thickness of the reaction layer is 30 µm.

### 3.2. Bond Analysis on the Ni-SiC/Sn5Sb3Ti Boundary

**Figure 8:**
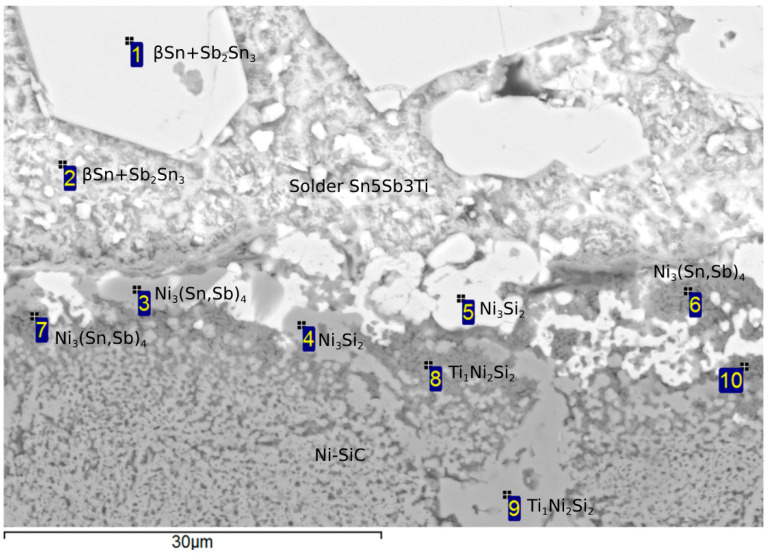
Point energy-dispersion analysis on the Ni-SiC/Sn5Sb3Ti boundary.

In previous studies [22,23,24,25] it has been suggested that Ti, as an active element, would be concentrated on the boundary of the composite Ni-SiC material, forming new phases. From the point EDX analysis (Figure 8), this assumption can be confirmed, since its concentration was detected in several spectra, as shown in Table 4. An increased Ni concentration on the boundary was also observed, which supports the formation of a reaction layer and, consequently, facilitates bond formation.

Figure 8 suggests that the solder matrix (representing Spectra 1 and 2) is formed of a peritectic mixture: βSn + Sb_2_Sn_3_. Spectra 3, 6, and 7, occurring on the Ni-SiC/solder boundary, corresponding to the Ni_3_(Sn,Sb)_4_ phase. Likewise, the Ni_3_Si_2_ phase occurs on the boundary, which is represented by Spectra 4 and 5. Therefore, one can say that an interaction between the composite material and solder took place. The distribution of the active Ti element on the Ni-SiC/solder boundary was studied via the occurrence of the Ti_1_Ni_2_Si_2_ ternary phase in Spectra 8 and 9. Spectrum 10 represents the Ni-SiC substrate.

**Figure 9:**
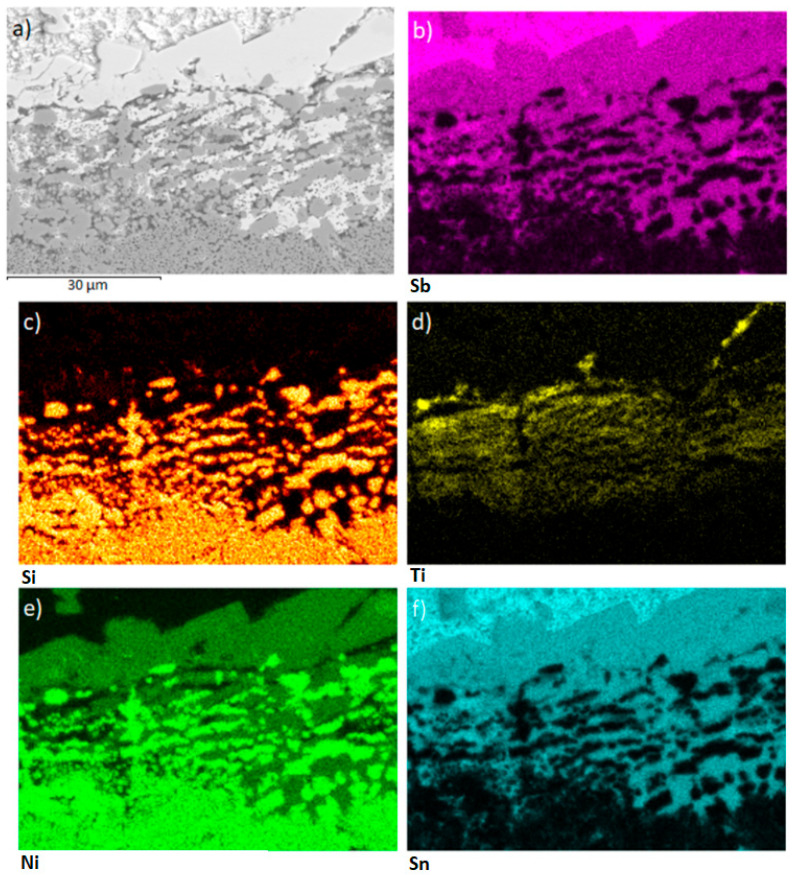
Planar distribution of elements on the boundary of the Ni-SiC/Sn5Sb3Ti joint: (**a**) SEM image of boundary; (**b**) Sb; (**c**) Si; (**d**) Ti; (**e**) Ni; (**f**) Sn.

**Figure 10:**
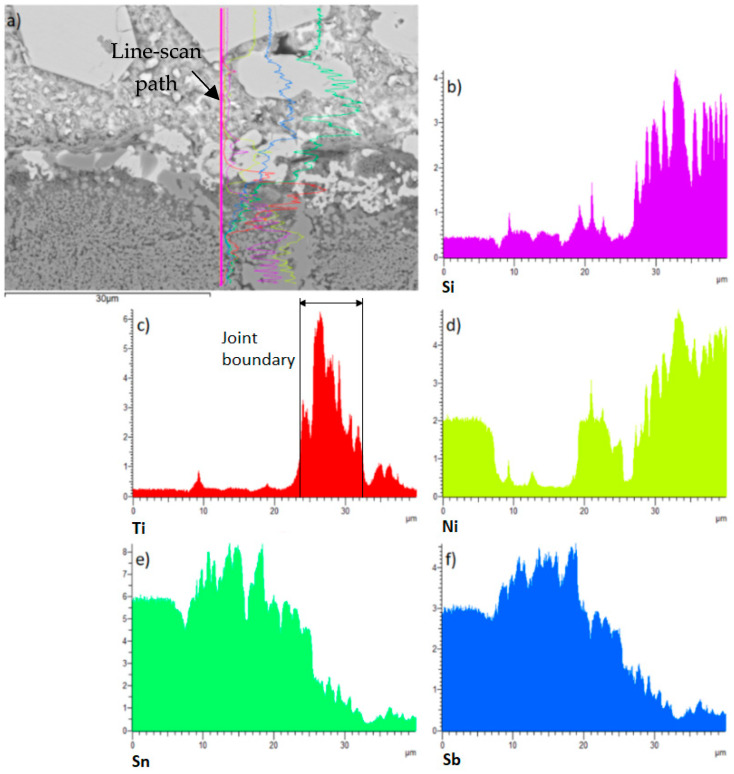
Line EDX analysis of the boundary in the Ni-SiC/Sn5Sb3Ti joint: (**a**) transition zone with a marked line; (**b**) Si; (**c**) Ti; (**d**) Ni; (**e**) Sn; (**f**) Sb.

Figure 9 shows the planar distribution of elements on the boundary of the Ni-SiC/Sn5Sb3Ti joint. The results of planar analysis suggest that the active Ti element significantly contributed to bond formation with the composite material. This statement is also corroborated by the line analysis shown in Figure 10. The concentration profile of the active Ti element (Figure 10c) represents the segregation of Ti on the boundary with the composite Ni-SiC material.

During the soldering process, the active Ti element was distributed from the solder to the boundary with the composite Ni-SiC material, with the formation of a transition zone ensuring the wettability of Ni-SiC. Wetting of the composite Ni-SiC material with the active solder type Sn5Sb3Ti takes place due to the formation of reaction products in the boundary.

### 3.3. Bond Analysis on the SiC/Sn5Sb3Ti Boundary

**Figure 11:**
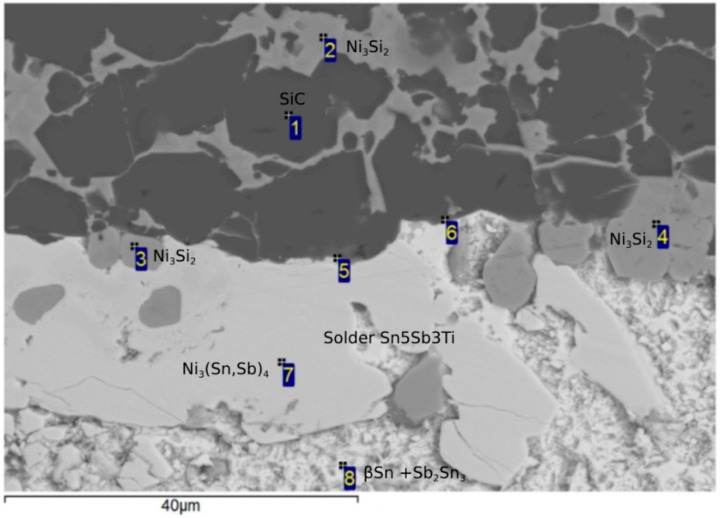
Point energy-dispersion analysis of the bond in the SiC/Sn5Sb3Ti boundary.

**Figure 12:**
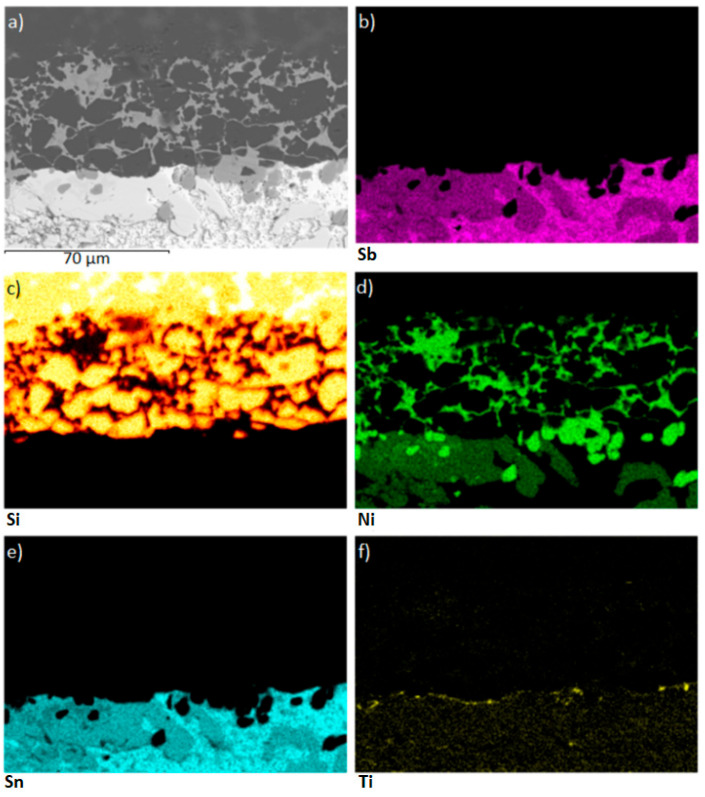
Planar distribution of elements in the boundary of the SiC/Sn5Sb3Ti joint: (**a**) SEM image of the boundary; (**b**) Sb; (**c**) Si; (**d**) Ni; (**e**) Sn; (**f**) Ti.

Figure 11 shows the microstructure of the boundary in the SiC/Sn5Sb3Ti joint. Spectrum 1 represents the SiC substrate. Spectra 2, 3, and 4 are formed from the Ni_3_Si_2_ phase with a high Ni content, which diffused from the Ni-SiC substrate. Interaction of the active component of the solder (Ti) was observed on the solder/substrate boundary in Spectra 5 and 6. Distribution of the active Ti element is also shown by the planar analysis of elements (Figure 12f). Also, Ni, which has diffused from the NiSiC substrate, was observed in the reaction layer. It was found that both these elements contribute to bond formation. Spectrum 7 is formed of the Ni_3_(Sn,Sb)_4_ phase, occurring in the solder proper. Spectrum 8 is formed of the βSn + Sb_2_Sn_3_ peritecticum. The results of the point EDX analysis are shown in Table 5.

**Figure 13:**
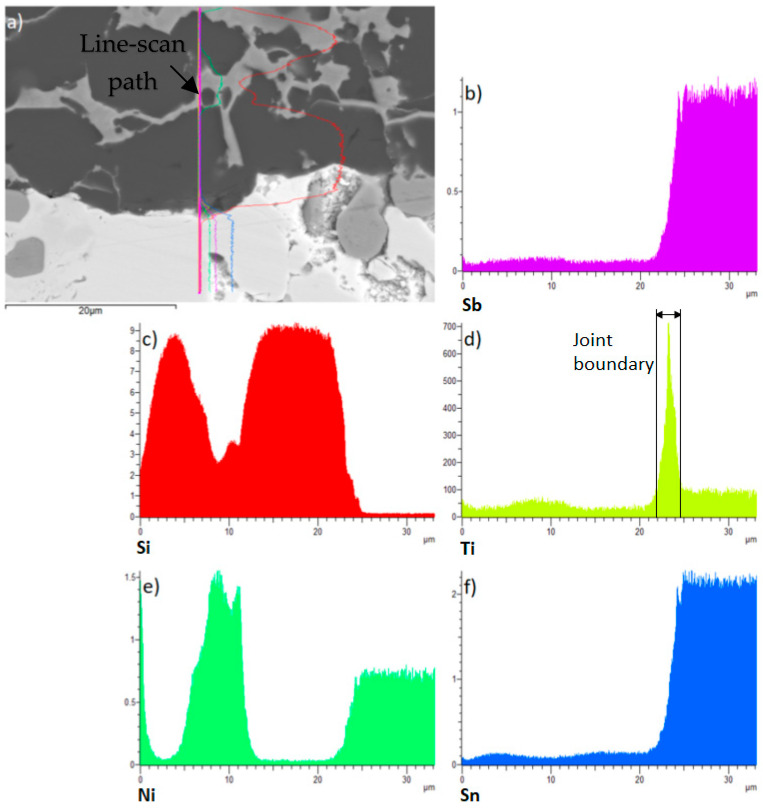
EDX analysis of the bond in the SiC/Sn5Sb3Ti boundary: (**a**) transition zone with a marked line; (**b**) Sb; (**c**) Si; (**d**) Ti; (**e**) Ni; (**f**) Sn.

During soldering, the active Ti element is distributed to the boundary with the ceramic SiC substrate via a diffusion mechanism, while a thin Ti reaction layer is observed in Figure 13f. This reaction layer has ensured the wetting of the SiC substrate and has thus supported bond formation. The line analysis in Figure 13d and the individual concentration profiles support this statement, as a yellow peak of titanium can be seen on the SiC/solder boundary.

### 3.4. Shear Strength of Soldered Joints

**Figure 14:**
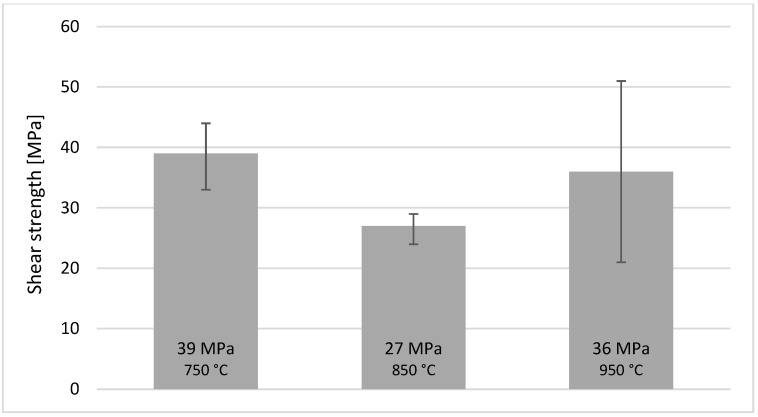
The shear strength of soldered joints of Ni-SiC/SiC fabricated using Sn5Sb3Ti solder, highlighting the dependence on soldering temperature.

Owing to the practical applicability of the Sn-5Sb-3Ti active solder, shear strength tests were carried out on three specimens at each soldering temperature (750, 850 and 950 °C). The results are summarised in Figure 14. Joints produced at 750 °C exhibited an average shear strength of 39 ± 6 MPa (33–46 MPa). Those soldered at 850 °C displayed the lowest strength, averaging 27 ± 2 MPa (25–29 MPa). Raising the temperature to 950 °C increased the average strength to 36 ± 15 MPa, with individual values ranging from 21 MPa to a maximum of 51 MPa, and the latter representing the strongest joint obtained in this study.

This drop-and-rebound trend is governed by the structure of the reaction layer and the residual stress state. At 850 °C, the Ti-rich intermetallic layer (Ti_6_(Sn,Sb)_5_ + Ni_3_(Sn,Sb)_4_) grows roughly twice as thick as at 750 °C, breaking up the ductile Sn–Sb matrix and building higher tensile stresses on cooling—hence the drop in strength. When the temperature reaches 950 °C, the layer becomes partly discontinuous and wetting improves; ductile Sn–Sb bridges now alternate with thinner brittle islands, cracks have a harder path, the stresses relax, and the average shear strength rises again.

**Figure 15:**
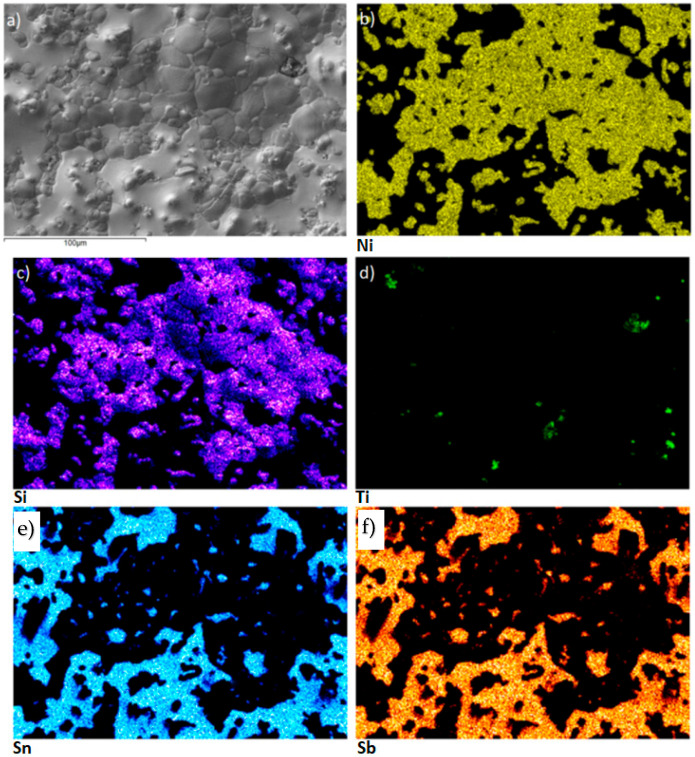
The fractured surface of the SiC/Sn5Sb3Ti/Ni-SiC joint on the SiC side and the planar distribution of individual elements: (**a**) fracture morphology; (**b**) Ni; (**c**) Si; (**d**) Ti; (**e**) Sn; (**f**) Sb.

The fracture mechanism of the lap-joint soldered at 950 °C (specimen that reached 51 MPa shear strength) was examined by scanning electron microscopy. The fracture surface on the SiC side of the SiC/Sn5Sb3Ti/Ni-SiC joint revealed that large areas of active solder remained attached to the ceramic, indicating that the crack propagated mainly through the ductile Sn-Sb matrix rather than along the solder–substrate interface. To better understand this fracture behaviour, planar EDS elemental mapping was performed on the same surface. The resulting maps are presented in Figure 15. The Si map (Figure 15c) highlights the locations in which the solder was torn away from the SiC substrate, confirming localised interfacial separation. This supports the conclusion that the fracture mechanism is primarily cohesive within the solder matrix rather than adhesive at the interface.

**Figure 16:**
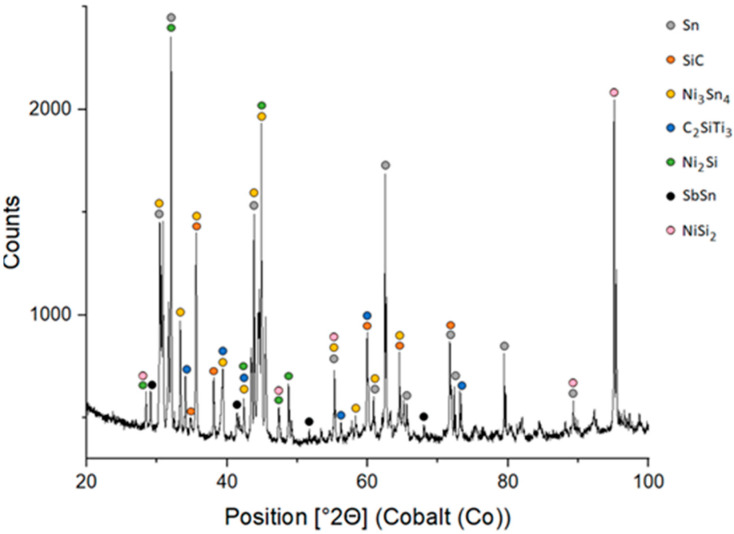
XRD pattern of the fractured surface of the SiC/Sn5Sb3Ti/Ni-SiC joint from the side of the SiC ceramic.

The fractured surface of the SiC/Sn5Sb3Ti boundary was studied through XRD analysis, the results of which are shown in Figure 16. The XRD analysis allowed for the identification of the Ni_3_Sn_4_, C_2_SiTi_3_, Ni_2_Si, SbSn and NiSi_2_ phases. Three of these, namely the C_2_SiTi_3_, Ni_2_Si and NiSi_2_ phase, enhance bond formation between the solder and the ceramic SiC substrate. The corresponding ICDD/ICSD reference codes for all identified phases are provided in Table 6.

## 4. Conclusions

The aim of this work was to study the combined soldered joints fabricated with ceramic SiC material and a Ni-SiC composite using the soldering alloy type Sn5Sb3Ti with electron beam heating. The primary goal of this work was to evaluate the wettability of an active solder on a composite substrate and the subsequent analysis of combined joints. The mechanical properties were studied with the shear strength test. The following results were achieved:

- The wettability experiment on the Ni-SiC composite started at a temperature of 750 °C. At this temperature, the solder did not wet the substrate surface and the wetting angle attained an average value of 94°. Increasing the temperature to 850 °C improved the wetting of the active solder, resulting in a reduction in the wetting angle to an approximate value of 60°. A significant improvement was achieved at 950 °C, with a wetting angle of 24° attained. These observations suggest that with increasing soldering temperature, the wetting angle of the active solder type Sn5Sb3Ti decreases.
- The specimen fabricated at a wetting temperature of 950 °C, where the smallest wetting angle was measured, was studied with SEM-EDX analysis. The Ni_3_(Sn,Sb)_4_ and Ti_6_(Sn,Sb)_5_ phases were observed on the Ni-SiC boundary. The reaction layer, formed of the Ti_6_(Sn,Sb)_5_ phase with an approximate thickness of 30 µm, provides evidence of Ti diffusion from the solder to the joint boundary and wetting of the composite substrate.
- A transition zone formed at the Ni-SiC/Sn5Sb3Ti boundary, where nickel migrated from the Ni-SiC composite into the solder via solid-state diffusion. The width of the Ni diffusion zone was approximately 10–25 µm, as shown by EDX line scans. No nickel diffusion into the SiC ceramic substrate was detected, confirming that migration was restricted to the interface. A similar effect was observed for Ti from the solder, with the distribution at the boundary confirmed by the presence of the Ti_1_Ni_2_Si_2_ phase. The bond at the SiC/Sn5Sb3Ti boundary is formed by the distribution of active Ti from the solder to the SiC ceramics, where a reaction layer forms and ensures wetting.
- The shear strength was measured on joints fabricated at three different soldering temperatures—750, 850 and 950 °C. Although the average shear strength at 950 °C (36 ± 15 MPa) was lower than that at 750 °C (39 ± 6 MPa), the highest individual shear strength of 51 MPa was recorded at 950 °C. This temperature also exhibited the greatest variability in mechanical performance, which may be attributed to local microstructural differences in the reaction layer formed at higher temperatures.
- XRD analysis of the fractured surface of the soldered joint from the side of the SiC ceramics showed the presence of the following phases: Ni_3_Sn_4_, C_2_SiTi_3_, Ni_2_Si, SbSn and NiSi_2_.

Future research will focus on evaluating joint durability under service conditions and scaling up the process to larger areas for industrial use.

## Figures and Tables

**Table 1:** The elemental composition of the Sn-Sb-Ti solder, as measured by ICP-AES.

Sample	Charge [wt %]			ICP-AES [wt %]		
	Sn	Sb	Ti	Sn	Sb	Ti
Sn5Sb3Ti	92.0	5.0	3.0	balance	5.18 ± 0.26	3.31 ± 0.34

**Table 2:** Parameters of electron beam soldering.

Accelerating voltage	55.0 kV
Current	10 mA
Focusing current	890 mA
Vacuum	1 × 10^−2^ Pa
Heating time	30 s
Heating temperature	750 °C, 850 °C, 950 °C
Time of cooling down	60 min.
Distance of jig surface from the electron gun	200 ± 1 mm

**Table 3:** The results from the point energy dispersion analysis on the boundary of the SnSb5Ti3/Ni-SiC joint.

Spectrum	C	Si	Ti	Ni	Sn	Sb	Phase
1	-	-	-	-	94.83	5.17	Peritectic βSn +Sb_2_Sn_3_
2	-	-	-	27.61	69.84	2.55	Ni_3_(Sn,Sb)_4_
3	-	-	-	26.56	70.39	3.05	Ni_3_(Sn,Sb)_4_
4	-	-	35.70	0.65	61.74	1.91	Ti_6_(Sn,Sb)_5_
5	-	-	2.88	0.94	92.17	4.01	Heterogeneous area
6	-	-	2.97	21.77	72.17	3.10	Heterogeneous area
7	27.88	14.12	-	57.99	-	-	Ni-SiC

**Table 4:** The results of the spot EDX analysis on the Ni-SiC/Sn5Sb3Ti boundary.

Spectrum	C	Si	Ti	Ni	Sn	Sb	Phase
1	-	-	-	-	96.09	3.91	Peritectics βSn + Sb_2_Sn_3_
2	-	-	-	-	95.37	4.74	Peritectics βSn + Sb_2_Sn_3_
3	-	-	-	39.63	56.30	4.07	Ni_3_(Sn,Sb)_4_
4	-	1.32	33.65	30.63	20.64	13.76	Ni_3_Si_2_
5	-	1.05	34.18	30.26	19.84	14.67	Ni_3_Si_2_
6	-	1.20	-	40.67	54.88	3.26	Ni_3_(Sn,Sb)_4_
7	-	2.87	1.48	40.68	52.55	2.42	Ni_3_(Sn,Sb)_4_
8	-	34.99	15.55	40.21	7.57	1.69	Ti_1_Ni_2_Si_2_
9	-	32.57	11.40	40.33	14.48	1.23	Ti_1_Ni_2_Si_2_
10	69.63	9.84	0.79	18.53	1.21	-	Ni-SiC

**Table 5:** The results of the point EDX analysis on the SiC/Sn5Sb3Ti boundary.

Spectrum	C	Si	Ti	Ni	Sn	Sb	Phase
1	44.44	53.56	-	0.63	1.37	-	Substrate SiC
2	-	25.62	-	74.38	-	-	Ni_3_Si_2_
3	-	22.59	-	77.41	-	-	Ni_3_Si_2_
4	-	22.83	1.29	74.73	1.15	-	Ni_3_Si_2_
5	-	27.97	8.46	15.41	44.80	3.36	Transition zone
6	-	49.81	1.08	2.15	44.93	2.03	
7	-	-	-	26.06	70.33	3.61	Ni_3_(Sn,Sb)_4_
8	-	-	-	-	93.57	6.43	Peritectic βSn + Sb_2_Sn_3_

**Table 6:** ICDD reference codes for crystalline phases identified by XRD in the Sn-5Sb-3Ti/Ni-SiC joint.

Ref. Code	Compound Name	Chem. Formula
03-065-2631	Tin	Sn
01-078-2780	Silicon Carbide	SiC
01-072-2569	Nickel Tin	Ni_3_Sn_4_
98-002-5762	Titanium Silicide Dicarbide	C_2_SiTi_3_
00-048-1339	Nickel Silicon	Ni_2_Si
00-033-0118	Antimony Tin	SbSn
03-065-2974	Nickel Silicon	NiSi_2_

## Data Availability

The original contributions presented in the study are included in the article; further inquiries can be directed to the corresponding author.
